# Antiviral activity of silymarin against chikungunya virus

**DOI:** 10.1038/srep11421

**Published:** 2015-06-16

**Authors:** Rafidah Lani, Pouya Hassandarvish, Chun Wei Chiam, Ehsan Moghaddam, Justin Jang Hann Chu, Kai Rausalu, Andres Merits, Stephen Higgs, Dana Vanlandingham, Sazaly Abu Bakar, Keivan Zandi

**Affiliations:** 1 Tropical Infectious Disease Research and Education Center (TIDREC), Department of Medical Microbiology, Faculty of Medicine, University Malaya, Kuala Lumpur, Malaysia; 2 Laboratory of Molecular RNA Virology and Antiviral Strategies, Department of Microbiology, Yong Loo Lin School of Medicine, National University of Singapore, Singapore; 3 Institute of Technology, University of Tartu, Tartu, Estonia; 4 Biosecurity Research Institute, Kansas State University, Manhattan, USA; 5 Department of Diagnostic Medicine and Pathobiology, Kansas State University, Manhattan, USA

## Abstract

The mosquito-borne chikungunya virus (CHIKV) causes chikungunya fever, with clinical presentations such as severe back and small joint pain, and debilitating arthritis associated with crippling pains that persist for weeks and even years. Although there are several studies to evaluate the efficacy of drugs against CHIKV, the treatment for chikungunya fever is mainly symptom-based and no effective licensed vaccine or antiviral are available. Here, we investigated the antiviral activity of three types of flavonoids against CHIKV *in vitro* replication. Three compounds: silymarin, quercetin and kaempferol were evaluated for their *in vitro* antiviral activities against CHIKV using a CHIKV replicon cell line and clinical isolate of CHIKV of Central/East African genotype. A cytopathic effect inhibition assay was used to determine their activities on CHIKV viral replication and quantitative reverse transcription PCR was used to calculate virus yield. Antiviral activity of effective compound was further investigated by evaluation of CHIKV protein expression using western blotting for CHIKV nsP1, nsP3, and E2E1 proteins. Briefly, silymarin exhibited significant antiviral activity against CHIKV, reducing both CHIKV replication efficiency and down-regulating production of viral proteins involved in replication. This study may have important consequence for broaden the chance of getting the effective antiviral for CHIKV infection.

Malaysia encompasses tropical rainforests and swamps, which are the niche for several mosquito-borne viruses. However, due to massive urbanization and deforestation several of these viruses were easily introduced to our human population including chikungunya virus (CHIKV). CHIKV is a member of *Togaviridae* family (genus Alphavirus) and causes chikungunya fever in humans. It was first isolated from the dengue-like outbreak in Tanzania (East Africa)^1^,^2^,^3^. Apart from the usual clinical manifestations such as fever, headache, lymphadenitis and rashes, the most prominent complaint from infected person is the chronic pain and stiffness due to arthritis or swelling of small joints which remains for weeks or even years, as define in the Makonde language in which ‘Chikungunya’ means ‘that which bends up,^4^,^5^,^6^.

Recently CHIKV infections emerged in Kenya and then were recorded in Comoros during 2004 with 5000 cases being reported. La’ Reunion in France (2005–2006), together with neighboring islands in the Indian Ocean, Seychelles, Madagascar, Mauritius and Mayotte; experienced an explosive outbreak with 300,000 cases and 237 deaths. CHIKV subsequently spread to India and Southeast Asia causing large-scale epidemics. In 2008, CHIKV was categorized as a Category C priority pathogen in the list of US National Institute of Allergy and Infectious Diseases (NIAID). To this date, more than 50 countries have been identified to be at risk^2^,^7^.

Phylogenetic analysis shows that CHIKV has three genotype variants; West African, East/Central/South African (ECSA) and Asian^8^. Malaysia had its first outbreak in late 1998 at the suburb Klang in the state of Selangor and the CHIKV was identified as Asian genotype. Localized outbreaks and clustered cases were reported in Larut Matang, Lama, Perak. The ECSA genotype virus caused outbreaks after 2006. In early 2008, together with the neighboring Singapore, the epidemic in Johor began. This spread throughout states in Peninsular Malaysia and in 2009, many cases occurred in Selangor, Melaka, Negeri Sembilan and Pahang states and peaked in Kelantan, Kedah, Terengganu, Perlis and Sarawak^4^,^9^,^10^. From April 2008 until March 2010, over 10,000 cases were reported in these nationwide outbreaks, although there were no fatalities^11^.

The main mosquito vectors for CHIKV are *A. aegypti* and *A. albopictus*^3^,^4^,^7^. *A. aegypti* was a primary vector for the outbreak that happened in Kenya, Comoros, Africa and Asia, but the ‘Asian Tiger Mosquitoe’ *A. albopictus* was the main vector for the Reunion and subsequent Indian Ocean outbreak^12^. Vector switching to *A. albopictus* became predominant due to the substitution of Alanine residue 226 to Valine residue in the CHIKV E1 protein (A226V)^13^. This convergent mutation seems to be due to the selective pressure as the impact of different and new environment or ecosystem.

Extensive studies continue around the world to find an effective cure, safe antiviral treatment or vaccine for CHIKV infection. In one study it was shown that chloroquine phosphate, an antimalarial drug, can decrease the intensity or duration of pain, and number of joints involved. However, the effectiveness of chloroquine to treat chronic symptoms could not be evaluated due to the lack of adequate control patients with these symptoms^14^. The synergistic effect of ribavirin with interferon alpha 2B against CHIKV infection was reported only in an *in vitro* study^6^. It has been shown that arbidol strongly interferes with the early stages of CHIKV infection by targeting the cellular membrane. However, it is only a potent inhibitor against *in vitro* CHIKV infection^15^.

Flavonoids are polyphenolic compounds that are present in different plants, foods and drinks. Flavonoids are well known for their different biological properties including: antimicrobial activity, anti-inflammatory activity, anti-allergic activity, and cytotoxic antitumor activity^16^. Quercetin has antiviral activity against herpes simplex virus (HSV) type-1, respiratory syncytial virus, pseudorabies virus, parainfluenza virus type 3 and Sindbis virus, a type member of genus *Alphavirus*. The mechanism of actions of quercetin includes: enhancing the antiviral activity of interferon, binding to viral proteins, and interfering with viral nucleic acid synthesis by binding to the viral polymerases. Another flavonoid, kaempferol is active against HSV, human coronavirus and rotavirus replication^17^.

Silymarin, extracted from milk thistle (*Silybum marianum*), was shown to inhibit hepatitis C virus (HCV) in both *in vitro* and *in vivo* by inhibiting HCV entry, RNA synthesis, viral protein expression and infectious virus production; in addition it also acts by blocking of the virus cell-to-cell spread^18^. It becomes our concern to explain the definition for the nomenclature of ‘milk thistle extract’, ‘silymarin’ and ‘silibinin’ since the findings that will be discussed further involve these three important terms. In brief, the initial extract of the crushed milk thistle seeds made up of 65–80% silymarin and 20–35% fatty acids, such as linoleic acid. Silymarin itself, is a complex of more than 7 flavonolignans (silybin A, silybin B, isosilybin A, isosilybin B, silychristin, isosilychristin and silydianin) and 1 flavonoid whereas silibinin is a semi-purified, commercially available fraction of silymarin, with an approximate 1:1 mixture of 2 diastereoisomeric compounds, silybin A and silybin B which are also referred to in the literature as silibinin A and B^19^.

Here we evaluated *in vitro* antiviral activity of quercetin, kaempferol and silymarin against a clinical isolate of CHIKV. Our results support the antiviral properties of silymarin as a promising compound for further investigations towards the development of an anti-CHIKV drug.

## Results

### Cytotoxicity of flavonoids

The MTS assay was used to determine cytotoxicity of each flavonoid for Vero and BHK cells. The CC_50_ value of each compound was calculated (Table 1). There was no observed cytotoxicity for cells treated with 0.1% DMSO (final concentration of solvent, used to dissolve flavonoids, in cell culture media).

### Antiviral activity assay

#### Primary screening

**Figure 1:**
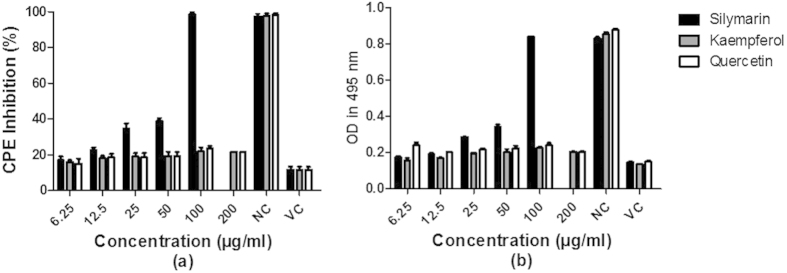
Silymarin inhibits CHIKV induced cytotoxic effects. CPE inhibition (**a**) and cell viability analysis by MTS assay (**b**) was carried out using Vero cells infected at an MOI = 1. Cells were treated with increasing concentrations (both kaempferol and quercetin concentration tested as high as 200 μg/ml) of indicated flavonoids and assayed 48 h post treatment. Results of panel (**a**) are normalized to respective values obtained for non-infected control cells which were set as 100% for each compound. CPE inhibition assay result shows that silymarin exhibits statistically significant (*P *< 0.05) dose-dependent inhibition on CPE presentation. Data from triplicate assays were plotted and analyzed from a Pearson two-tailed test (Graph Pad Prism Version 5, Graph Pad Software Inc., San Diego, CA).

Different non-cytotoxic concentrations of silymarin, kaempferol and quercetin were tested on CHIKV-infected Vero cells to find the effective compound. A CPE inhibition assay was used at 48 hpi. It was shown that 100 μg/ml of silymarin significantly inhibited the CHIKV-CPE presentation (*P* = 0.0007) (Fig. 1a). This finding was not unexpected as silybin (also known as silibinin) is one of the components of silymarin and has been shown to possess anti-CHIKV activity^20^. In contrast, the highest concentrations of quercetin and kaempferol only exhibited 25% CPE inhibitory activity (Fig. 1a). The results of CPE inhibition assay were further confirmed by using MTS assay (Fig. 1b).

### Silymarin inhibits early post-entry stages of CHIKV replication

From analyzed compounds only silymarin was able to suppress CHIKV mediated cytotoxicity. Therefore it was chosen for a time-of-addition assay performed with aim to determine which stage of virus infection cycle this compound affected.

**Figure 2:**
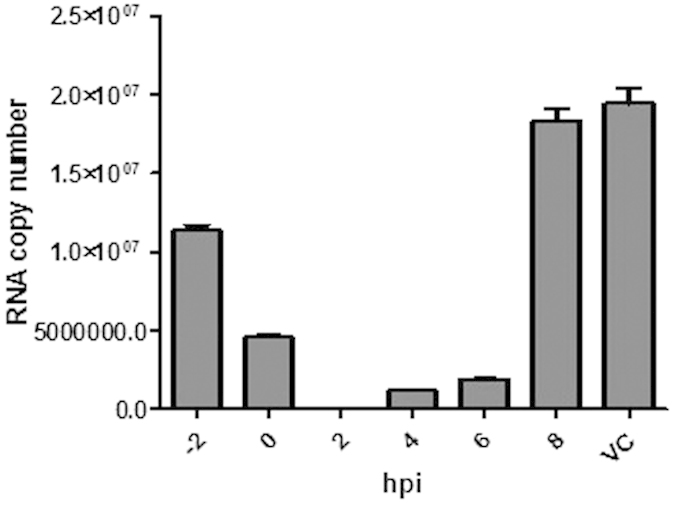
Silymarin inhibits early post entry stages of CHIKV replication cycle. The Vero cells were treated with 100 μg/ml of silymarin at stated time-points and infected with CHIKV at an MOI = 1 (“0” time point). Copy number of viral RNA in supernatants at was measured at 48 h pi. Using qRT-PCR. Data from triplicate assays were plotted using Graph Pad Prism Version 5 (Graph Pad Software Inc., San Diego, CA). Error bars represent standard deviation. VC- vehicle control (cells treated with media containing 0.1% DMSO only).

**Figure 3:**
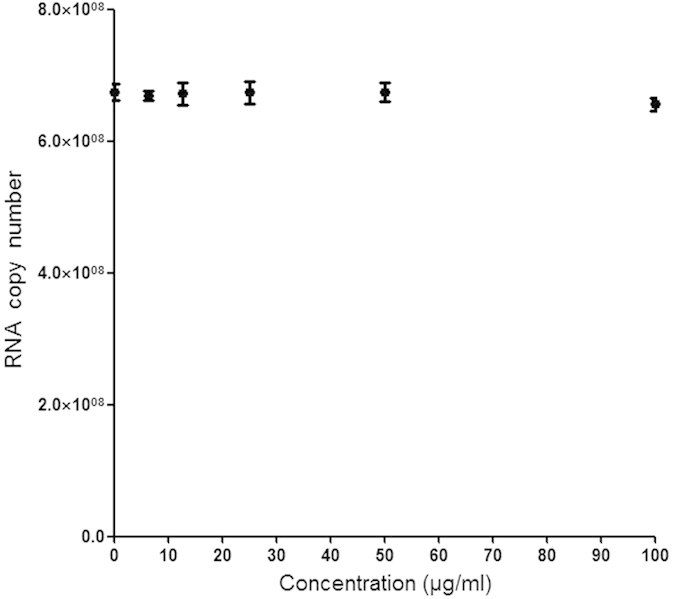
Effect of silymarin against CHIKV cell entry. Silymarin does not exhibit statistically significant (*P *> 0.05) dose-dependent inhibition against CHIKV internalization to the Vero cells. Vehicle–treated (0.1% DMSO) cells were used as control (“0”) concentration). Data from triplicate assays were plotted and analyzed from a non-parametric correlation (Spearman) two-tailed test (Graph Pad Prism Version 5, Graph Pad Software Inc., San Diego, CA).

As it is shown in Fig. 2 the inhibition was most efficient when silymarin was added at 2  hpi. The time-dependence of inhibitory effect of compound is coherent with hypothesis that its anti-CHIKV activity may be due inhibition of some early, but probably post-entry, step of CHIKV replication cycle. Anti-entry assay has also been performed and the result has clearly shown that there is no antiviral activity of silymarin against CHIKV at the anti-entry phase (Fig. 3).

### Evaluation of antiviral effect of silymarin using CHIKV replicon cell line

**Figure 4:**
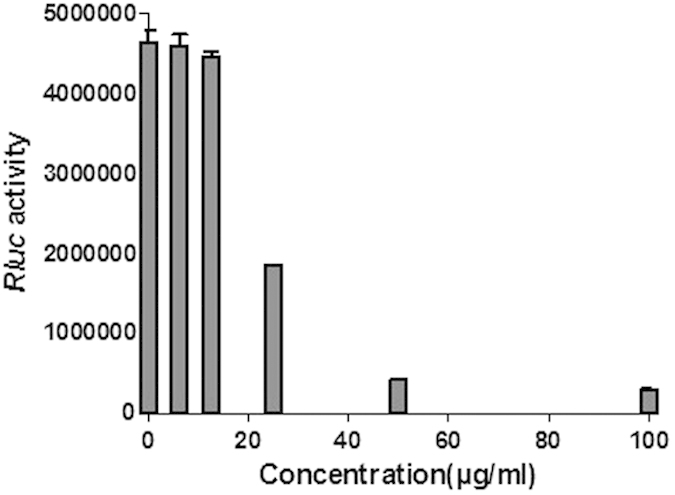
Evaluation of anti-CHIKV activity of silymarin using BHK-CHIKV-NCT replicon cell line. Silymarin exhibits a statistically significant (*P* < 0.05) dose-dependent inhibition on the Rluc activity produced by BHK-CHIKV-NCT replicon. The Rluc activity was measured at 48 h post treatment. Vehicle–treated (0.1% DMSO) cells were used as control (“0”) concentration). Data from triplicate assays were plotted and analyzed from a non-parametric correlation (Spearman) two-tailed test (Graph Pad Prism Version 5, Graph Pad Software Inc., San Diego, CA).

The CHIKV replicon cell line contained non-cytotoxic replicon of CHIKV which, in addition to the virus replicase proteins, expresses also puromycin acetyl transferase, *EGFP* and *Rluc* markers^20^. In this system the *Rluc* activity is proportional to viral replicon RNA replication. It was found that 100 μg/ml of silymarin suppressed the activity of *Rluc* marker expressed by the CHIKV replicon by 93.4% (Fig. 4). The inhibition was highly significant (*P* = 0.016) confirming that silymarin affects post-entry steps of CHIKV infection. This data also indicates that silymarin can interfere with CHIKV RNA replication by affecting the viral replicase system. Interestingly, in previous study^20^ IC_50_ of silybin, one of the major components of silymarin, was estimated as 59.8 μM (approximately 30 μg/ml). Compared to this silymarin was somewhat more efficient as nearly three-fold inhibition was observed at 25 μg/ml indicating that other components of silymarin likely contributed to its anti-CHIKV activity.

### Silymarin interferes with post-entry stages of CHIKV infection in a dose dependent manner

**Figure 5:**
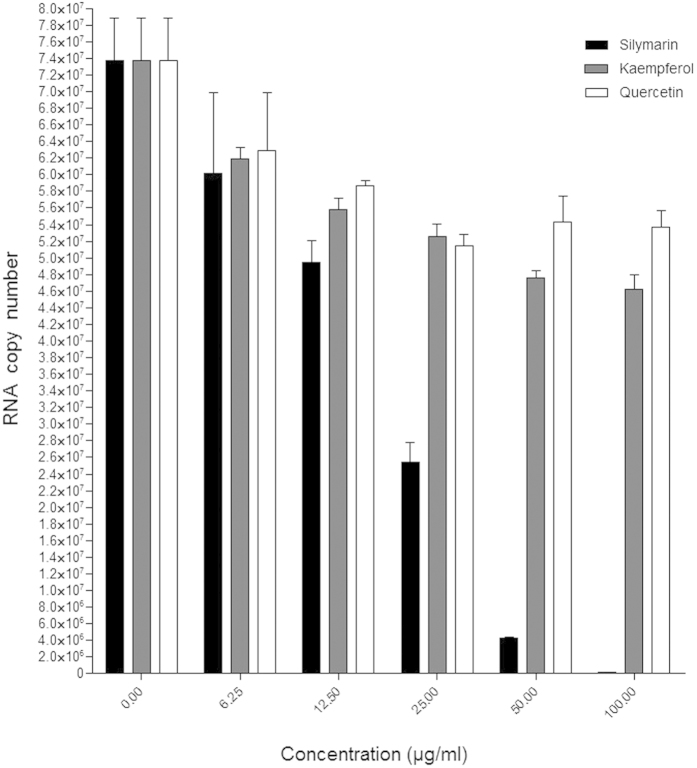
Silymarin reduces CHIKV RNA copy number in growth medium in dose dependent manner. Silymarin exhibits a statistically significant (*P *< 0.05) dose-dependent inhibition of CHIKV RNA levels. As it is shown in the figure, there is no significant inhibitory effect from quercetin and kaempferol against CHIKV intracellular replication (*P *> 0.05). Statistical significance is analyzed from a Pearson two-tailed test (Graph Pad Prism Version 5, Graph Pad Software Inc., San Diego, CA).

To confirm the post-entry antiviral activity of silymarin against CHIKV a virus yield assay using qRT-PCR was used. The CHIKV RNA copy number in supernatants of cells treated with each concentration of inhibitor and that from the control cells was interpreted and depicted (Fig. 5). There is a significant reduction of CHIKV replication as treated with increasing concentrations of silymarin (*P* = 0.0199), as compared to the vehicle control treated cells. Based on this data it was calculated that silymarin had IC_50_ =  16.9 μg/ml with Selectivity Index (SI) of 25.1. Taking into account that adding silymarin 2 hpi showed significant inhibitory effect against CHIKV replication confirms that silymarin acts at the post-entry stage of CHIKV infection. In contrast, in similar assay neither kaempferol nor quercetin showed any significant inhibitory effect against CHIKV replication (Fig. 5). As it is shown in Fig. 5, 100 μg/ml of silymarin can inhibit the CHIKV yield by more than 99%.

**Figure 6:**
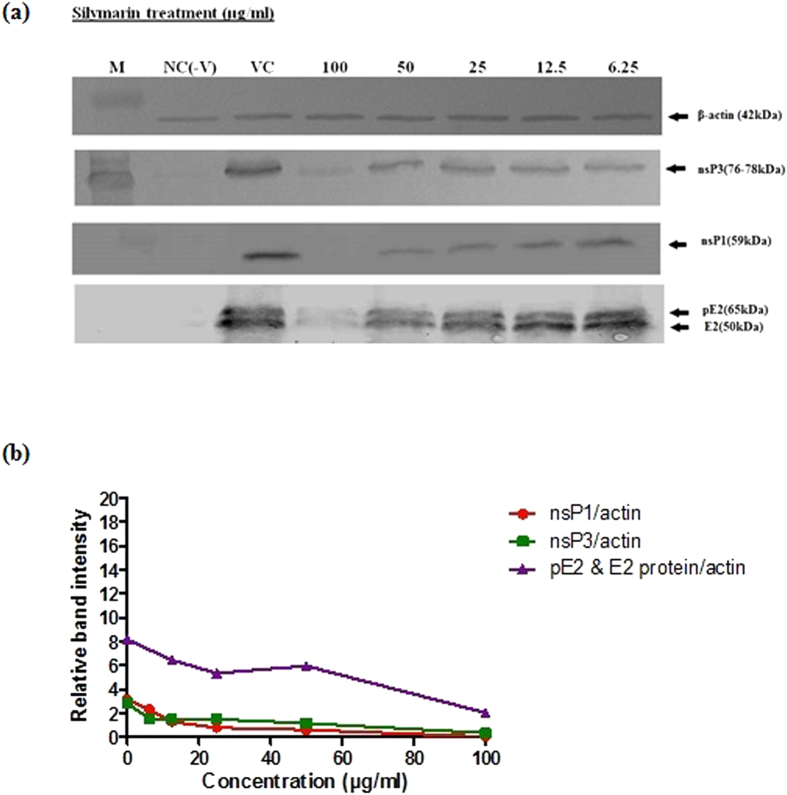
Silymarin suppresses accumulation of CHIKV-encoded proteins. Western blot analysis was performed to determine the effect of silymarin on production of CHIKV nsP1, nsP3 and E2 proteins. (**a**) Bands of the specific proteins as observed on the blot. A dose dependent reduction of CHIKV nsP1, nsP3 and E2 proteins were observed upon silymarin treatments for 48 h. (**b**) The above mentioned observation confirmed by calculating the relative band intensity (calculated from the band intensity of sample/band intensity of β-actin loading control) using Image J. β-actin is used as a loading control for each set of samples.

To evaluate whether the silymarin also affects the CHIKV protein synthesis, a series of western blot analyses were performed using lysates of the CHIKV infected and silymarin treated Vero cells (Fig. 6). As it is evident from constant levels of β actin, silymarin treatment did not result in protein degradation and, at used concentrations, the compound was not toxic for the Vero cells. In contrast, dose-dependent reduction of amounts of nsP1, nsP3 and E2 proteins was observed (Fig. 6) indicating that silymarin limited CHIKV replication and virus-encoded protein synthesis within the treated cells.

### Silybin interferes with early post-entry of CHIKV infection in dose-dependent manner

Questioned by whether the silymarin effects on the CHIKV replication are influenced by the fractions it is made up of, since the study done by Pohjala L. *et al.*, 2011 has proved that the silybin (one of the silymarin fraction) can suppressed the activities of *Rluc* marker gene expressed by the CHIKV replicon, we have evaluated the effect of silybin against CHIKV intracellular replication. Hence, silybin was tested for its early post-entry activity and we confirmed its anti-CHIKV activity in a dose-dependent manner with the IC_50_ of 69.7 µg/ml, which is higher than the IC_50_ of silymarin. This result makes us to conclude that the activity of silymarin against CHIKV replication might also be enhanced by other components or fractions that made it up.

## Discussion

Considering the crippling pains that persist for weeks and even years due to CHIKV infections and the absence of antiviral treatment for the virus, the search for effective antiviral compounds is imperative. Due to their known broad spectrum anti-viral activity and low toxicity, we evaluated three flavonoids as viable candidate compounds^21^. From these compounds only silymarin was identified as the flavonoid with significant anti-CHIKV activity. It was considered that it has potential for future development as its use results in ≥70% reduction of CHIKV infection based on CPE inhibition and MTS assays (Fig. 1). Remarkably, the effective concentration of silymarin was much lower than MNTD. In contrast, neither kaempferol nor quercetin showed significant antiviral activity against *in vitro* replication of CHIKV in both assays.

To quantify the copy number of viral RNA released (most probably in form of genomic RNA packed into virions) from infected cells were used qRT-PCR and amplified part of nsP3 encoding region of CHIKV genome. Since nsP3 which is involved in the negative-strand and subgenomic RNA synthesis, the quantification of the negative-strand RNA reflects the active viral replication, we selected the nsP3 as the gene target for qRT-PCR to quantify the virus load during replication^22^,^23^,^24^,^25^. Our data demonstrated that treatment with silymarin results in effective reduction of number of released viral genome indicating reduction of viral RNA synthesis and/or virion formation and release.

In post-adsorption assay silymarin taken at 100 μg/ml resulted in ≈99% inhibition of CHIKV replication. We therefore performed a time-of-addition assay using this concentration of silymarin to determine the stage of infection where the silymarin treatment results in biggest antiviral effect. The result was consistent with our data from post-adsorption assay; hence it can be concluded that silymarin is most effective if it is added shortly after virus infection (2 hpi). The reason for lower anti-CHIKV activity of pretreatment with silymarin could be due to the half-life of silymarin which based on previous bioavailability study is less than 4 hours^26^, although, there is no data available on *in vitro* half-life or stability of silymarin which might be interesting for future studies.

As our findings were consistent with hypothesis that silymarin effect on post-entry stages of CHIKV dose response analysis was also performed using a CHIKV replicon cell line where no virus entry or exit takes place. Again, it was found that silymarin is able to suppress the activity of *Rluc* marker expressed by CHIKV replicon. This finding is consistent with that from previous assay and confirms that silymarin suppresses CHIKV RNA replication and does it in dose-dependent manner. The *Rluc*, expressed by CHIKV replicon, is fused to nsP3 protein of the virus. Accordingly, reduction of *Rluc* activity indicates reduced amounts of nsP3-*Rluc* protein in CHIKV replicon cell line.

To further confirmation of anti-CHIKV activity of silymarin, we have shown the effect of silymarin on viral protein synthesis in CHIKV infected cells (Fig. 5). Again decrease of viral protein synthesis was observed. This effect could be due to inhibition of CHIKV RNA replication and/or transcription. However, as in virus expression and replicon cell lines the synthesis of viral RNAs and proteins are coupled further study is necessary to evaluate the direct effect of silymarin on inhibition of newly synthesized CHIKV proteins. It is possible that some of non-structural proteins of CHIKV represents direct target for silymarin. The possibilities include nsP1 protein, which is involved in the synthesis of the negative strand of viral RNA and RNA capping, and nsP3 protein, that is another component of the viral replicase complex. Down regulation of E2 expression may represent consequence of suppression of replication (directly or via inhibition of ns-protein(s)). However, this down regulation is clearly relevant from point of view of development of effective antiviral as E2 protein is one of the important virion glycoproteins and is essential for receptor binding. Interestingly, a previous study showed that silymarin can inhibit the expression of NS5B of hepatitis C virus (HCV) which is catalytic subunit of HCV replicase^26^. Although, the hepatoprotective property of silymarin in the context of blocking HCV infection based on *in vivo* studies is another important criteria for this compound to be considered as a therapeutic candidate for hepatitis C^19^,^27^,^28^.

This study represents an important first report on the anti-CHIKV properties of silymarin. Previously other flavonoids such as apigenin, chrysin, naringenin and silybin, which are also one of components of silymarin, are known to be inhibitors of viral replication mainly through affecting important cellular elements for CHIKV replication^20^. However, inhibitory properties of silymarin are somewhat different from that of silybin. Thus our data for silymarin warrants further mechanistic studies to characterize the antiviral properties of all its components and other flavonoid compounds and head-to-head comparison of their antiviral effects at lower concentrations/ different conditions so that the best option of anti-CHIKV can be determined.

## Conclusion

In summary, our study showed that silymarin exhibited significant *in vitro* antiviral activity against CHIKV. It suppressed post-entry stages of viral replication, most likely CHIKV RNA replication significantly with a dose dependent manner. Coherent with this the expression of proteins, needed for RNA replication, and also expression of viral structural E2 protein were down-regulated. In contrast to silymarin it was found that quercetin and kaempferol are unable to suppress CHIKV replication and accordingly are not good candidates for anti-CHIKV drugs. These findings warrant future mechanistic, *in vivo* anti-viral, toxicity and pharmacokinetic studies as part of the process for evaluation of silymarin as a potential anti-CHIKV therapeutic.

## Materials and Methods

### Virus and cells

The CHIKV isolate used in this experiment was a clinical isolate from an outbreak in Johor in 2008 coded as MY/065/08/FN295485. It belongs to the ECSA genotype and has the A226V mutation in E1 protein^29^,^30^. BHK-21 (baby hamster kidney) and Vero (African green monkey kidney) cells from the American Type Culture Collection (ATCC) were used in this study. Vero cells have been widely used in the *in vitro* antiviral research for example in the antiviral testing for the chikungunya virus, human enterovirus 71 and Coxsackievirus A16^31^,^32^. Both cell lines were cultured using Eagle’s Minimum Essential Medium (EMEM, Gibco, NY, USA) containing 10% inactivated fetal bovine serum (FBS) and penicillin-streptomycin, and incubated at 37°C with 5% CO_2_.

The CHIKV MY/065/08/FN295485 strain was propagated in BHK-21 cells and harvested after full cytopathic effect (CPE) was observed. Virus stock titer was determined by the tissue culture infectious dose 50 (TCID_50_) methods^33^; then the obtained stock was aliquoted and stored at –70 °C. During the time of virus propagation and antiviral assay the FBS concentration of the cell culture medium was reduced to 2%. Vero cell line was used for further experiments such as MTS assay and antiviral assays.

The CHIKV replicon cells^20^ were grown and maintained in Dulbecco’s Modified Eagle’s Medium (DMEM, Gibco, NY, USA) supplemented with 8% fetal bovine serum (FBS), 2% tryptose-broth phosphate and penicillin-streptomycin, and incubated at 37°C with 5% CO_2_.

### Flavonoids

Silymarin, quercetin and kaempferol were purchased from Sigma-Aldrich Corporation (Sigma-Aldrich, St. Louis, MO, USA). Silybin was purchased from Selleckchem Corporation (Selleckchem, TX, USA). The stock solutions of flavonoids, 50 μg/ml, were prepared in dimethyl sulfoxide (DMSO) (Sigma-Aldrich, St. Louis, MO, USA) and stored in −20 °C for future use. Stock solution was diluted with EMEM and was filtered through a syringe filter with 0.2 μm pore size (Millipore, MA, USA) at the time of use; DMSO concentration in working solutions prepared on EMEM was kept at 0.1%.

### Cell viability assay

The MTS (3-(4,5-dimethylthiazol-2-yl)-5-(3-carboxymethoxyphenyl)-2-(4-sulfophenyl)-2H-tetrazolium) assay (Promega, WI, USA) was performed to evaluate the cytotoxicity of tested compounds against Vero cells and BHK21 cells according to the manufacturer’s protocol. Briefly, monolayers of Vero cells were grown in 96-well plate and were treated with different concentrations of each compound in triplicate together with negative control (media containing 0.1% DMSO). The plate was then incubated at 37 °C with 5% CO_2_ for 48 hours before the MTS assay was performed. Treated and control cells were kept for two days at 37 °C, under similar conditions and duration until used for antiviral activity assay. After two days post-treatment, MTS solution was added to the cells and incubated for 4 hours at 37 °C with 5% CO_2_ prior to absorbance detection at 495 nm wavelength using Infinite 200 Pro multiplate reader (Tecan, Männedorf, Switzerland). All experiments were conducted in triplicate. The half maximal cytotoxic concentration (CC_50_) for each compound was determined through this assay using Graph Pad Prism 5 (Graph Pad Software Inc., San Diego, CA, USA, 2005).

### Antiviral activity assays

#### Primary assay for antiviral activity

A monolayer of Vero cells were grown in 96-well plate in EMEM containing 2% inactivated FBS. The tested compounds were added to the wells in triplicate together with CHIKV at an MOI =  1. The plate was then incubated at 37°C with 5% CO_2_ for 48 hours. The assay was conducted in duplicate for each concentration of each compound. After two days, the plate was viewed under the microscope and the degree of cytopathic effect (CPE) as measure of virus replication inhibition was expressed as the percent yield of virus control (% virus control = CPE experimental group/CPE virus control × 100). The results were confirmed by performing the MTS assay (Promega, WI, USA) according to the manufacturer’s protocols. All experiments were repeated three times independently. The statistical analysis on percentage of CPE inhibition was performed by using the Gaussian populations (Pearson) two-tailed assay.

### Anti-entry assay

The procedure of the anti-entry assay was modified and performed according to Lee RCH *et. al*., 2013^34^. Monolayers of Vero cells were grown in 96-well plate with EMEM supplemented with 10% inactivated FBS. The Vero cells were then infected with CHIKV and the plate was incubated for 1 hour at 4 °C. Non-adsorbed virus was then washed with 1xPBS. Tested compound was added in different concentrations and incubated at 37 °C with 5% CO_2_ for 2 hours. The plate was again washed with 1xPBS and treated with citrate buffer (pH = 3) to inactivate the non-internalized virus, before the plate was again washed with 1xPBS. The EMEM supplemented with 2% inactivated FBS was added into every wells and the plate was incubated for 48 hours at 37 °C with 5% CO_2._

### CHIKV replicon cell line based assay

Monolayers of CHIKV replicon cells were prepared in 96-well white plate (Corning Inc, NY, USA) and treated with different concentrations of tested compounds. After 48 hours incubation at 37°C with 5% CO_2_ the activity the *Renilla* Luciferase (*Rluc*), expressed by CHIKV replicon, was detected using *Renilla* luciferase assay (Promega, WI, USA) performed according to the manufacturer’s protocols. The luminescence signal was then measured using the GloMAX 20/20 Luminometer (Promega, WI, USA), plotted against the log transformation of the concentrations of compounds and a sigmoidal curve fit with variable slope was created to obtain the half maximal inhibitory concentration (IC_50_) value for each compound by Graph Pad Prism 5 (Graph Pad Software Inc., San Diego, CA, USA, 2005). Data were represent as the means ± standard error of the mean (SEM) from triplicate assay from three independent experiments. The statistical analysis to determine the correlation between the concentration of silymarin and the *Rluc* activity was performed by using non-parametric correlation (Spearman) two-tailed assay.

### Time-of-addition assay

In this experiment, a monolayer of Vero cells were grown in 96-well plate in EMEM containing 2% inactivated FBS. The wells (in triplicate) were designated as −2, −1, 0, 2, 4, 6 and 8-hour, that they are named according to the time of CHIKV (MOI = 1) infection. As for −2 hour, Vero cells were treated with 100 μg/ml of silymarin. The plate was then incubated for one hour at 37 °C with 5% CO_2._ One hour later, −1 hour wells were also treated with 100 μg/ml of silymarin. At the 0 hour, all wells except the control wells were infected with CHIKV and again incubated for one hour at 37 °C with 5% CO_2._ After 2-hour incubation and for every 2-hour after incubation at 37 °C with 5% CO_2,_ 100 μg/ml silymarin were added to −2, 4, 6 and 8 hour wells respectively. The plate was then incubated at 37 °C with 5% CO_2_ for 48 hours.

The supernatant of the treated and control wells (treated with 0.1% DMSO was used as vehicle control) were collected two days post-treatment and analyzed using qRT-PCR. The half maximal effective concentration (EC_50_) of silymarin was calculated using Graph Pad Prism 5 (Graph Pad Software Inc., San Diego, CA, USA, 2005). All data are from triplicate assay from three independent experiments.

### Quantitative reverse transcription PCR(qRT-PCR)

A qRT-PCR assay was used to quantify the CHIKV RNA copy number. For this amplification of 136 base region of nsP3 encoding sequence was performed as described by Chiam and colleagues^35^. The primers were nsP3-F(5’-GCGCGTAAGTCCAAGGGAAT-3’) and nsP3-R (5’-AGCATCCAGGTCTGACGGG-3’). The cDNA was first generated from the RNA extracted from the supernatants (QIAGEN, Germany) of the previous assays plate, using nsP3-R primer and Superscript III Reverse Transcriptase (Life Technologies, USA) according to the manufacturer’s protocol. The unincorporated primers were then digested with 20U of Exonuclease I (New England Biolabs, USA). The qRT-PCR was performed with a Step-OnePlus Real-Time PCR System (Life Technologies, USA) with 2 **× **Power SYBRGreen PCR Master Mix (Life Technologies, USA), following the manufacturer’s protocol, and using serially diluted standards. Cycling parameters were 95 °C for 10 min, 40 cycles of 95°C for 15 s and 60 °C for 1 min. The amplified product was verified by melting curve analysis. The statistical analysis to determine the correlation between the RNA copy number and the silymarin concentration was performed by using the Gaussian populations (Pearson) two-tailed assay.

### Western blotting

Vero cells at the density of 3 × 10^6^ cells were seeded into a 75 cm^2^ flask in EMEM containing 2% FBS and penicillin-streptomycin. The next day, each flask was infected with CHIKV inoculum at an MOI = 1, placed on a rocker for 30 minutes, and then incubated at 37 °C with 5% CO_2_ for 2 h. Then each flask was treated with different concentrations (100, 50, 25, 12.5, 6.25 μg/ml) of the trial compound, control flasks were treated with solutions containing 0.1% DMSO. All the flasks were then incubated at 37 °C with 5% CO_2_ until the appearance of CPE in the vehicle control. Once CPE was observed, cells were scraped, washed with PBS and lysed using 300 μl of 1% Triton X100 (Sigma-Aldrich, St. Louis, MO, USA) containing complete protease inhibitor cocktail (Sigma-Aldrich, St. Louis, MO, USA) at 4 °C for 45 min. Cellular debris was pelleted out by centrifugation at 16,000 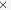
g for 5 min. A Micro BCA^TM^ Protein Assay Kit (Thermo Scientific, Rockford, IL) was used to quantify the protein concentration for each sample. Lysate containing 100 μg of protein were denatured using SDS-loading buffer and proteins were separated using SDS-PAGE in 12% gels. The gels were then equilibrated in Towbin buffer (0.025 M Tris, 0.192 M glycine 20% methanol) for 10 min and proteins were transferred to a PVDF membrane using the Bio-Rad wet transfer system (Bio Rad, San Francisco, CA). For detection of nsP1 and nsP3, membranes were blocked with 1X PBS 1% Casein Blocker (Bio Rad, San Francisco, CA) for 1 h at room temperature on a shaker. The blots were rinsed three times with 1X PBS Tween20 before being incubated with primary anti-CHIKV nsP1, anti-CHIKV nsP3 or anti-CHIKV E2 rabbit polyclonal antibodies in 1% casein solution. The blots were then washed three times with 1X PBS Tween20 for 15 min each time. This was followed by incubation with the secondary goat anti-rabbit IgG (Abcam, Cambridge, UK) antibodies conjugated with horseradish peroxidase (HRP) for 1 h at room temperature on an orbital shaker. Membranes were washed three times with PBS containing Tween 20 for 15 minutes each time. For the loading control, separate blots containing the same samples were incubated with primary anti-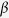-actin mouse monoclonal antibody conjugated with HRP (Cell Signaling Technology, MA, USA) dissolved in 1% Casein for 1 hour at room temperature on shaker. The blots were then washed three times with 1X PBS Tween 20 for 15 minutes each time. Membranes were developed by the colorimetric method using appropriate substrates (Thermo Scientific, Rockford, IL).

## Additional Information

**How to cite this article**: Lani, R. *et al.* Antiviral activity of silymarin against chikungunya virus. *Sci. Rep.* **5**, 11421; doi: 10.1038/srep11421 (2015).

## Figures and Tables

**Table 1:** Cytotoxicity of flavonoids against Vero cells and BHK-21 cells.

**Compounds**	**Vero cells**		**BHK-21 cells**	
	**CC50 (μg/ml)**	**MNTD (μg/ml)**	**CC50 (μg/ml)**	**MNTD (μg/ml)**
Silymarin	425.1	>200	305.4	>150
Quercetin	>1000	>400	1665	>500
Kaempferol	>1000	>400	537.3	>200
Silybin	>200	>50	NA*	NA*

^*^NA: not applicable.

MTS assay was used to evaluate the cytotoxicity of the compounds. Different concentrations of tested flavonoids up to 800 μg/ml were used to treat the Vero cells and BHK-21 cells for 2 days. The maximum non-toxic dose (MNTD) of silymarin as the effective compound against CHIKV is >200 μg/ml (on Vero cells) and >150 μg/ml (on BHK21 cells) as more than 95% of the treated cells are viable.
